# Role of Interleukin-10 and Abdominopelvic Ultrasound as a Potential Predictor of Disease Severity in Dengue Hemorrhagic Fever

**DOI:** 10.7759/cureus.5249

**Published:** 2019-07-27

**Authors:** Ambreen Tauseef, Farhat Ijaz, Farid Ahmad Chaudhary, Zaima Ali, Tanzeela Akram, Rana Khurram Aftab, Gulfam Ahmad

**Affiliations:** 1 Department of Physiology, Combined Military Hospital Lahore Medical College and Institute of Dentistry, Lahore, PAK; 2 Department of Cardiothoracic and Vascular Surgery, Rehmatul Lil Alameen Post Graduate Teaching Institute of Cardiology, Lahore, PAK; 3 Department of Physiology, Lahore Medical & Dental College, Lahore, PAK; 4 Department of Physiology, King Edward Medical University, Lahore, PAK; 5 School of Medical Sciences, Faculty of Medicine and Health, The University of Sydney, Sydney, AUS

**Keywords:** il-10, dengue hemorrhagic fever, abdominopelvic ultrasound, outcome

## Abstract

Introduction

Dengue viral infections are a major cause of morbidity and mortality in tropical/subtropical countries. Early and prompt detection of dengue hemorrhagic fever (DHF), though challenging, is helpful to identify an individual that would benefit from intensive therapy.

Objective

The goal of this study was to determine the plasma interleukin-10 (IL-10) levels in DHF patients at four to seven days of disease onset and 24 hours after the first sample. We also aimed to determine the association of plasma IL-10 levels and abdominopelvic ultrasound findings.

Methods

A total of 50 registered DHF patients aged 15 to 50 years were recruited. Plasma IL-10 concentration measurements and abdominopelvic ultrasounds were performed. Patients were also categorized based on ultrasound grading I to IV (based on severity). Outcomes were described as recovery and shock. Platelet count and hematocrit percentages were also recorded.

Results

Plasma IL-10 levels were elevated in DHF patients and associated with fatal outcomes (p = 0.00). Binary regression-coefficient showed the direct effect of high levels of plasma IL-10 on the fatal outcome of patients 24 hours after the first sample (p = 0.04). Disease severity was predicted by a positive correlation between ultrasound grades and outcomes (p = 0.00). Spearman’s correlation coefficient found a highly significant inverse relationship between plasma IL-10 levels and platelet count after 24 hours (p = 0.01). However, a significant positive relationship was observed between elevated plasma IL-10 levels and hematocrit percentage after 24 hours (p = 0.01).

Conclusion

Elevated plasma IL-10 levels and abdominopelvic ultrasonography are promising potential predictors of disease progression and fatal outcome in DHF patients.

## Introduction

Dengue virus infection is the most important arthropod-borne viral disease, affecting tropical and subtropical regions around the world [1,2]. The incidence of dengue disease has increased 30-fold in the last 50 years [3]. The four serotypes of dengue virus are capable of causing a broad spectrum of clinical disease starting from mild illness such as dengue fever, to a life-threatening condition known as dengue hemorrhagic fever (DHF), which may further progress to fatal shock (dengue shock syndrome (DSS)) [4]. According to the World Health Organization 2011 revised classification, dengue fever is “dengue without warning signs” whereas DHF and DSS are “dengue with warning signs” and “severe dengue,” respectively [5]. Patients may develop warning signs of severe disease around the time the fever begins to subside (usually four to seven days after symptom onset) [6].

The underlying pathogenesis of dengue viral disease is still unknown and controversial [7]. However, it mainly results from the imbalance of the host immune system [8]. Moreover, the interplay of the inflammatory response and deregulated cytokine production in DHF may play a key role in protection or increased disease severity [9]. Among cytokines, interleukin-10 (IL-10) known to be a major regulator in the inflammatory process by possessing a dual nature of both an anti-inflammatory agent and immunosuppressant [10].

According to previous studies, elevated levels of IL-10 correspond with the third to the seventh day of the illness (the defervescence phase), suggesting its positive correlation with the disease severity [11,12]. Nevertheless, abdominopelvic ultrasound emerged as an important adjunct to clinical and laboratory profiles in diagnosing and predicting the severity of DHF. In this study, we evaluated the role of IL-10 and abdominopelvic ultrasound against the outcomes of patients and as a potential predictor of disease severity in DHF.

## Materials and methods

After receiving Institutional Ethical Review Board approval, we received informed written consent from all the participants. A total of 50 registered patients diagnosed with DHF were included from all the major hospitals. The study included patients aged 15 to 50 of both genders. We divided all patients into four groups depending on their presentation on different days of their course of disease, within four to seven days of their disease onset. We excluded patients diagnosed with dengue disease, Chikungunya, Congo hemorrhagic fever, hepatitis B and C, malaria, enteric fever, diabetes mellitus, or any other malignancy. DHF diagnosis was based on immunoglobulin M (IgM)-positive for dengue virus, increases in hematocrit with a decrease of >10,000 platelets/mm^3^ in 24 hours compared to previous measurement or concurrent with platelet counts ≤ 100,000/mm^3^. We retrieved 3mL of venous paired blood samples aseptically; first, at the time of presentation (within four to seven days of onset) followed by a second blood sample retrieved 24 hours after the first sample. Plasma was separated by centrifugation, aliquoted, labeled, and kept frozen at -20°C until further analysis for IL-10 levels by enzyme-linked immunosorbent assay. A consultant radiologist performed abdominopelvic ultrasounds for all patients, and findings such as thickened gall bladder wall, pleural effusion, and ascites were noted.

The data were entered and analyzed by using IBM SPSS Statistics for Windows, Version 20.0 (IBM Corp., Armonk, NY, USA). All quantitative variables were expressed as mean ± SD, whereas qualitative variables were described using frequencies and percentages. Shapiro-Wilk test was applied for normality. For comparison in four groups, we used Kruskal-Wallis test, and for comparison between two independent groups, Mann Whitney-U test. For related samples, we applied the Wilcoxon Signed-Rank test. The effect of plasma IL-10 on the outcome of patients was related by binary logistic regression. Spearman’s correlation coefficient was referred to see the relationship of plasma IL-10 with platelet count and hematocrit percentage. A p-value ≤ 0.05 was considered statistically significant.

## Results

This study included 50 subjects, divided into four groups according to their days of presentation within four to seven days of their disease onset. They consisted of 38 (76%) male patients with a mean age of 31 ± 11 years and 12 (24%) female patients with a mean age of 27 ± 9 years. Forty-six of 50 patients (92%) with a mean body temperature of 29.8°C ±10.7°C were afebrile and presented on the fifth and sixth days of dengue illness (during the defervescence phase; p = 0.006; Table 1). 

**Table 1:** Distribution of febrile and afebrile patients by day of fever at the admission time (four to seven days after onset of disease) *P < 0.05

Day of fever at admission time	Fever		Afebrile		Total		χ^2^	P-value
	n	Percent	n	Percent	n	Percent		
4^th^ day	4	100%	9	19.5%	13	100%	12.38	.006*
5^th ^day	0	0%	15	32.6%	15	100%		
6^th ^day	0	0%	15	32.6%	15	100%		
7^th ^day	0	0%	7	15.2%	7	100%		
Total	4	100%	46	100%	50	100%

**Figure 1:**
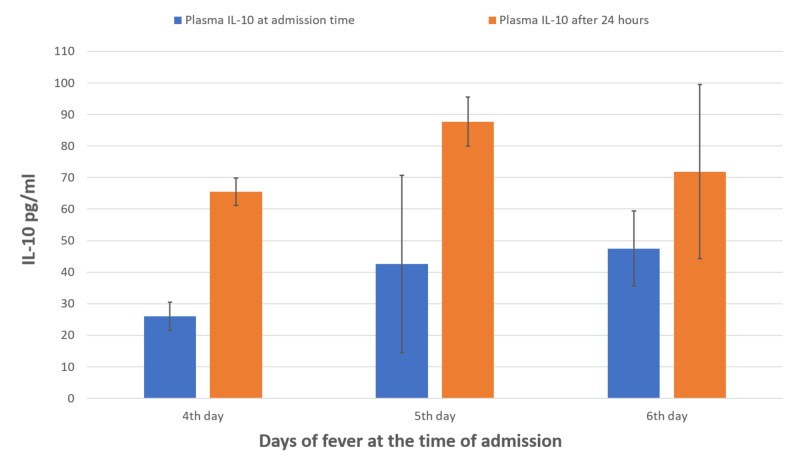
Mean levels of plasma IL-10 at the time of admission and after 24 hours of shocked patients (four to six days)

**Figure 2:**
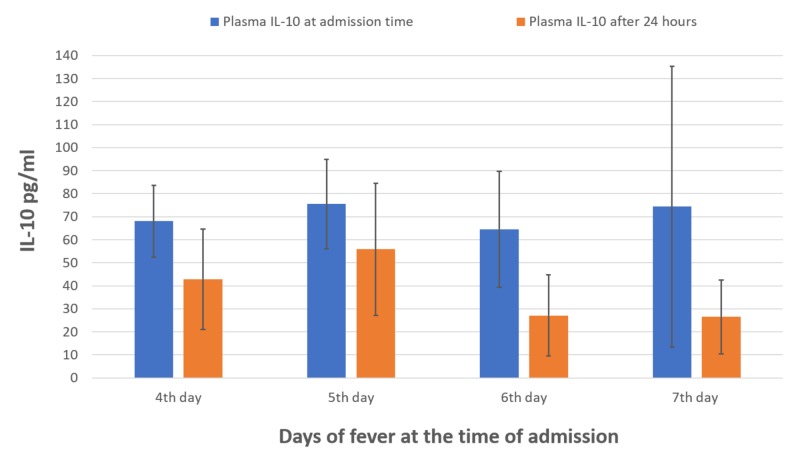
Mean levels of plasma IL-10 at the time of admission and after 24 hours of recovered patients (four to seven days)

Similarly, seven (14%) patients followed shock on the fifth and sixth days. Mean plasma IL-10 levels were elevated in all the patients suffering from DHF, however, in shocked patients (n=7), the levels were markedly raised after 24 hours (mean, 87.6 ± 7.7 pg/ml) compared levels at time of admission (mean, 42.5 ± 28.0 pg/ml; Figure 1). In recovered cases, the levels of plasma IL-10 were decreased after 24 hours (mean, 26.5 ± 16.0 pg/ml) as compared to elevated levels at the time of admission (mean, 74.3 ± 61.6 pg/ml; Figure 2). 

Most patients had grades II, III, and IV severity (χ2 = 9.44, p = 0.024) on abdominopelvic ultrasound (Table 2). We noted a significant difference between grades II and grade IV severity in platelet count after 24 hours (p = 0.007). We observed a significant difference in hematocrit percentage found in grades I, II, and IV at the time of admission (p = 0.001) and after 24 hours (p = 0.000). For plasma IL-10, a significant difference was only present after 24 hours between grades I and IV (p = 0.012), grade II and IV (p = 0.006) and in grade III and IV (p = 0.040; Table 3).

**Table 2:** Distribution of patients by grading of ultrasound and gender Chi-square likelihood ratio, 9.44 *P < 0.05 a: Gallbladder wall thickening b: Grade I + pelvic ascites (mild) / fluid in hepatorenal pouch / hepatomegaly / right-sided pleural effusion c: Grade I / II + pelvic ascites (moderate) d: Grade III + abdominopelvic ascites / bilateral pleural effusion

Grading of ultrasound	Male		Female		Total		χ^2^	P-value
	n	Percent	n	Percent	n	Percent		
I^a^	6	16%	1	8%	7	14%	9.44	0.024*
II^b^	19	50%	6	50%	25	50%		
III^c^	4	11%	5	42%	9	18%		
IV^d^	9	24%	0	0%	9	18%		
Total	38	100%	12	100%	50	100%

**Table 3:** Mann Whitney-U test for pair-wise comparison for grades of ultrasound *P < 0.05, significant Abbreviation: Hct, hematocrit.

Variables	Ultrasound	n	Mean Rank	Sum of Ranks	Mann-Whitney U test	P-value
Hct after 24 hours	I	7	4.50	31.50	3.50	0.001*
	IV	9	11.61	104.50		
IL-10 levels after 24 hours	I	7	5.21	36.50	8.50	0.012*
	IV	9	11.06	99.50		
Platelets after 24 hours	II	25	20.20	505.00	45.00	0.007*
	IV	9	10.00	90.00		
Hct at admission time	II	25	14.86	371.50	46.50	0.008*
	IV	9	24.83	223.50		
Hct after 24 hours	II	25	13.64	341.00	16.00	0.000*
	IV	9	28.22	254.00		
IL-10 levels after 24 hours	II	25	14.78	369.50	44.50	0.006*
	IV	9	25.06	225.50		
IL-10 levels after 24 hours	III	9	6.94	62.50	17.50	0.040*
	IV	9	12.06	108.50		

We noted a statistically significant association between severity of ultrasound grades and outcome (χ2 = 26.01; p = 0.000). Of 43 recovered patients, 25 showed findings followed by grade II, and of seven shocked patients, six showed findings consistent with grade IV (Table 4).

**Table 4:** Association between grading of ultrasound and outcome Chi-square likelihood ratio, 26.01. *P < 0.05, significant

Grading of Ultrasound	Outcomes		Total	χ^2^	P-value
	Recovered	Shock		26.01	0.000*
I	7	0	7		
II	25	0	25		
III	8	1	9		
IV	3	6	9		
Total	43	7	50		

We applied binary logistic regression to find the effect of IL-10 on the outcome of patients, showing the positive association of increased levels of plasma IL-10 on fatal outcomes (1.072 with 95% CI, p = 0.004).

Spearman’s correlation coefficient was used to see the relationship of plasma IL-10 with platelet count and hematocrit percentage. We noted a highly significant reverse relationship between plasma IL-10 levels and platelet count after 24 hours. Similarly, a significant positive correlation was observed between plasma IL-10 and hematocrit percentage after 24 hours (rho = 0.452, p = 0.01; Table 5).

**Table 5:** Correlations of plasma IL-10 levels with platelet count and hematocrit %, at the time of admission and after 24 hours *Correlation is significant at the 0.01 level (2-tailed). Abbreviation: Hct, hematocrit; rho, correlation coefficient.

Variables	Platelets at admission time (rho)	Platelets after 24 hours (rho)	Hct at admission time (rho)	Hct after 24 hours (rho)	P-value
IL-10 levels at admission time	-.105	-.115	.113	-.055	0.011
IL-10 levels after 24 hours	-.199	-.405(*)	.212	.452*	0.011

## Discussion

Our findings positively correlate increased plasma concentration of IL-10 with disease severity in DHF patients. In patients who suffered shock, plasma IL-10 levels increased significantly24 hours after admission. In patients who recovered later, IL-10 plasma levels decreased 24 hours after admission. In this study, overall raised levels of plasma IL-10 align with previous reports [13,14]. In a study conducted in Cuba, IL-10 levels were observed to be higher and positively correlated with disease progression in severe dengue infections [15]. Similarly, Gurugama et al. and Ubol et al. highlighted the role of elevated levels of IL-10 in the pathogenesis of DHF [16,17].

During the course of the disease, DHF/DSS occurs on the third to the seventh day of illness. The fifth and sixth days of the fever are the most vulnerable days to develop shock. Moreover, peak levels of plasma IL-10 have been noted on the fifth and sixth days of fever [18]. In the present study, most patients presenting on the fifth and sixth days of illness were afebrile on admission (representing their defervescence phase) with significantly high levels of plasma IL-10. Plasma leakage and the outcome of dengue disease is mostly defined during the phase of defervescence. Therefore, a positive correlation of the elevated level of plasma IL-10 with disease severity requires special attention during this phase.

To strengthen our conclusion about the predictive role of IL-10 in DHF, we correlated the levels of IL-10 with grades (based on severity) of ultrasound. Higher IL-10 levels associated positively with the degree of plasma leakage. We found a strong positive association between the severity of ultrasound grades and fatal outcomes (p = 0.000). Patients with significantly higher levels of plasma IL-10 and shock also tended to have grade III and IV in abdominopelvic ultrasound. This is likely because cytokines cause a malfunction of vascular endothelial cells rather than the destruction of the small vessels, which aligns with a study conducted in 2010 by Srikiatkhachorn et al. [19]. Srikiatkhachorn suggested IL-10 is a potential marker of severe disease as its measured levels are correlated positively with the size of pleural effusion in the defervescence phase [19].

This study found a significant positive correlation between high levels of plasma IL-10 and hematocrit percentage (p = 0.000), likely due to the positive correlation of elevated IL-10 levels with the degree of plasma leakage. A higher level of plasma leakage seems directly related to an increased hematocrit. However, we found a highly significant reverse relationship between increasing levels of plasma IL-10 and platelet count (p = 0.000). This phenomenon might be because raised plasma IL-10 correlates positively with hemorrhagic manifestations and platelet decay.

This correlation of IL-10 with disease severity is not only due to its immunosuppressive nature, but also due to its ability to down-regulate antigen-presenting cell response and suppress the intracellular antiviral response, which facilitates dengue virus replication [20]. However, the results of this study contrast with studies that suggest raised levels of IL-10 could be in disagreement with the pathogenic role ascribed to this cytokine [21]. Similarly, Perez et al. suggested that the low levels of IL-10 seem to increase the susceptibility to DHF [22].

As the full burden of disease severity cannot be attributed to a single cytokine, a limitation of this study was the inability to study a range of cytokines due to cost restrictions. We recommend further studies with larger sample sizes be conducted using sequential measurements in all four days of DHF for additional insights into this disease.

## Conclusions

This study observed a predictive role of plasma IL-10 and a positive correlation of its elevated levels with the degree of plasma leakage in the defervescence phase of DHF. Moreover, an increase in IL-10 concentrations along with a decrease in platelet and white blood cell count is associated with an increased probability of DHF severity. Hence, prompt determination of plasma IL-10 levels may serve as a useful indicator for the early diagnosis of potential DHF cases for better clinical intervention resulting in decreased mortality and morbidity.
